# Recreational marathon running does not cause exercise-induced left ventricular hypertrabeculation

**DOI:** 10.1016/j.ijcard.2020.04.081

**Published:** 2020-09-15

**Authors:** Andrew D'Silva, Gabriella Captur, Anish N. Bhuva, Siana Jones, Rachel Bastiaenen, Amna Abdel-Gadir, Sabiha Gati, Jet van Zalen, James Willis, Aneil Malhotra, Irina Chis Ster, Charlotte Manisty, Alun D. Hughes, Guy Lloyd, Rajan Sharma, James C. Moon, Sanjay Sharma

**Affiliations:** a Cardiology Clinical & Academic Group, St George's University of London, London, UK; b Institute for Cardiovascular Science, University College London, London, UK; c Barts Heart Centre, St Bartholomew's Hospital, London, UK; d Guy's and St Thomas' NHS Foundation Trust, London, UK; e East and North Hertfordshire NHS Trust, Stevenage, UK; f Royal United Hospitals Bath NHS Foundation Trust, Bath, UK; g Infection and Immunity Research Institute, St George's, University of London, London, UK

## Abstract

**Background:**

Marathon running in novices represents a natural experiment of short-term cardiovascular remodeling in response to running training. We examine whether this stimulus can produce exercise-induced left ventricular (LV) trabeculation.

**Methods:**

Sixty-eight novice marathon runners aged 29.5 ± 3.2 years had indices of LV trabeculation measured by echocardiography and cardiac magnetic resonance imaging 6 months before and 2 weeks after the 2016 London Marathon race, in a prospective longitudinal study.

**Results:**

After 17 weeks unsupervised marathon training, indices of LV trabeculation were essentially unchanged. Despite satisfactory inter-observer agreement in most methods of trabeculation measurement, criteria defining abnormally hypertrabeculated cases were discordant with each other. LV hypertrabeculation was a frequent finding in young, healthy individuals with no subject demonstrating clear evidence of a cardiomyopathy.

**Conclusion:**

Training for a first marathon does not induce LV trabeculation. It remains unclear whether prolonged, high-dose exercise can create de novo trabeculation or expose concealed trabeculation. Applying cut off values from published LV noncompaction cardiomyopathy criteria to young, healthy individuals risks over-diagnosis.

## Highlights

• Athletes often show excessive ventricular trabeculation.
• It is unknown whether left ventricular noncompaction cardiomyopathy can be acquired.
• It is proposed that trabeculation may result from athletic remodeling to exercise.
• Imaging is prone to overdiagnosis of left ventricular noncompaction cardiomyopathy.
• Recreational marathon running does not increase left ventricular trabeculation.

## 1 Introduction

Cross sectional studies have highlighted that athletes often show increased left ventricular (LV) trabeculation in comparison to non-athletes, particularly competitive soccer and rugby players [1] though the estimated prevalence varies widely across different athlete populations and using different definitions, from 1.4% [2] to 18.3% [1].

It has been proposed that exercise can induce acquired LV trabeculation in potentially predisposed individuals, which risks phenotypic overlap with possible cardiomyopathy. Some have proposed integrating other functional parameters in an attempt to distinguish between benign and pathological excessive LV trabeculation [3,4]. However, longitudinal evidence demonstrating a cause and effect relationship between exercise and increased LV trabeculation has been lacking. The Journal previously published a case series of four basketball players where training and detraining resulted in changes in trabeculation, though the definition of LV hypertrabeculation and quantification results were not reported [5].

In a separate population, 25% of primagravida women developed LV trabeculation during pregnancy, which resolved in the majority of women post-partum. The authors proposed that this was likely an epiphenomenon resulting from cardiovascular adaptation to the loading conditions of pregnancy, where a 50% rise in blood volume increases cardiac preload [6].

Building on this work, we hypothesized that young, novice marathon runners can develop exercise-induced LV trabeculation as a result of their preparatory training. Marathons are popular mass-participation endurance events, where participants of varying athletic backgrounds increase their physical activity levels to meet a personal goal. This provides an opportunity to study a natural experiment of short-term cardiovascular remodeling in response to running training. We undertook a prospective, longitudinal study to investigate if typical marathon training for first-time participants could induce an increase in LV trabeculation assessed by both echocardiography and cardiac magnetic resonance (CMR). For clarity we use the term “excessive trabeculation” to describe subjects where published thresholds for left ventricular non-compaction (LVNC) criteria are exceeded.

## 2 Methods

Subjects were considered for inclusion if they were aged 18–35 years old and had never run a marathon distance previously. Individuals were excluded if they had pre-existing cardiovascular disease during preliminary investigations or contraindication to cardiovascular magnetic resonance (CMR). Novice marathon runners were identified through the database records of the organizers (Virgin Money London Marathon) and received notification of the study through e-mail advertisement. Interested runners made contact through a call center, were subsequently contacted by telephone and recruited if fulfilling inclusion criteria.

Subjects were encouraged to follow a beginner's training plan, consisting of approximately 3 runs per week, increasing in difficulty over a 17-week period leading up to the London Marathon race [7].

Upon notification of their awarded places, 68 first-time marathon runners underwent echocardiography, CMR and cardiopulmonary exercise testing on a semi recumbent cycle ergometer 186 ± 4 days (mean ± standard deviation) before and 16 ± 4 days after the marathon. Detailed information on study participant recruitment, methods, baseline characteristics and cardiovascular remodeling findings have been published previously [8]. Imaging studies were analyzed by an accredited, experienced cardiologist (A.D.), blinded to subject identity and time point.

Statistical analyses were performed with R version 3.3.0 (R Project for Statistical Computing). Data were tested for normality with the Shapiro-Wilk test and assessed in histograms. Normally distributed data are presented as mean ± SD and skewed data are presented as median with inter-quartile range. Differences between group means were compared using a paired *t-*test, if parametric, or Wilcoxon signed rank test if non-parametric. Differences in paired categorical data (positive and negative LV non-compaction criteria) were compared using McNemar's test. Reproducibility of measurements between raters was assessed with two-way, mixed single measures intraclass correlation coefficient (ICC) analysis for absolute agreement. Statistical significance was defined as a two-tailed value of *P* < 0.05.

## 3 Results

Subjects were aged 29.5 ± 3.2 years, 53% were male and 90% of white European ethnicity with a median self-reported baseline physical activity level of 2.0 h per week (range 0–10 h, IQR 1.5–2.5 h). Subjects achieved a median race finish time of 04:31:00 (HH:MM:SS, range 02:56:10–06:51:20), which was comparable with London Marathon general race participants. Mean peak oxygen consumption was 40.5 ml/kg/min (100% predicted) in men and 35.2 ml/kg/min (115% predicted) in women at baseline and did not change after the marathon. No definite evidence of cardiomyopathy was detected in any subject and cardiac dimensions were within normal range.

**Fig. 1:**
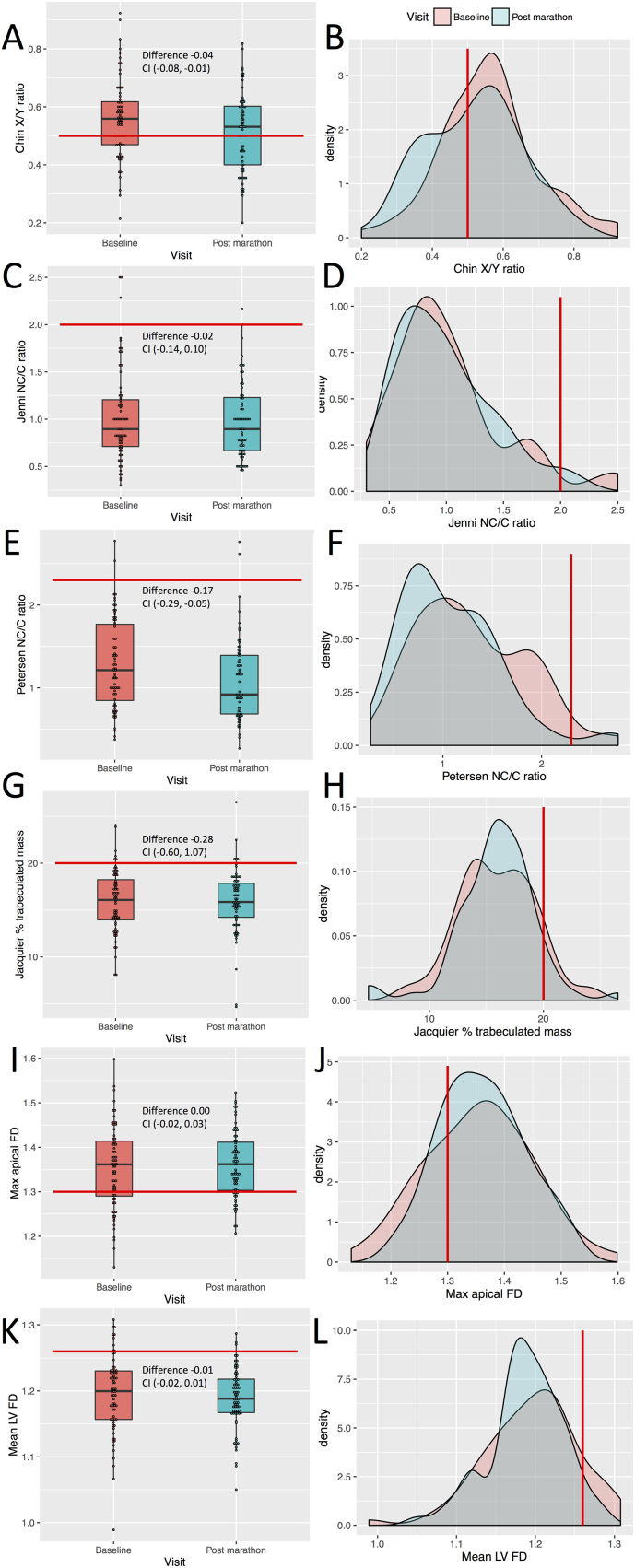
Comparison of changes in left ventricular trabeculation indices. Boxplots (left) and density curves (right) indicate trabeculation measurements at baseline (pink) and post marathon (blue). Trabeculation indices measured were Chin X/Y ratio (A & B), Jenni NC/C ratio (C & D), Petersen NC/C ratio (E & F), Jacquier percentage trabeculated mass (G & H), Captur maximal apical (I & J) and mean global fractal dimensions (K & L). Red lines mark the published diagnostic thresholds for suspected cases of left ventricular non-compaction. Included in the boxplots is the mean difference with 95% confidence intervals. CI, confidence interval; FD, fractal dimension; LV, left ventricle; Max, maximum. (For interpretation of the references to color in this figure legend, the reader is referred to the web version of this article.)

**Fig. 2:**
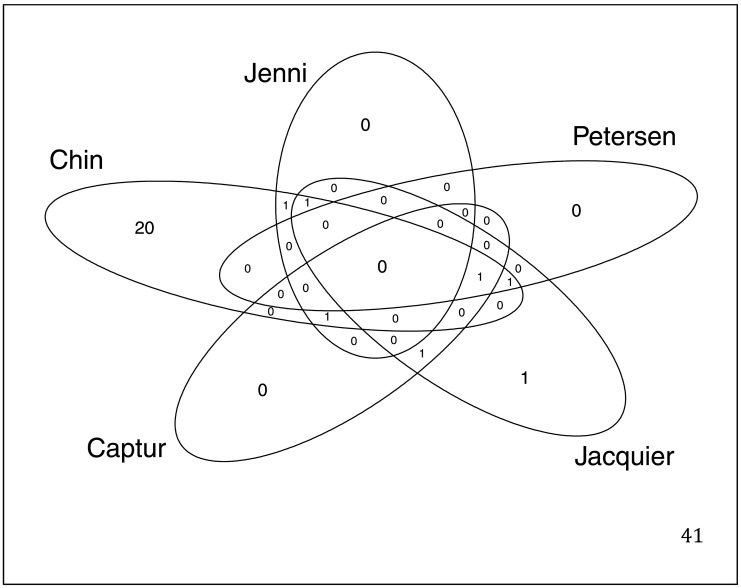
Venn diagram of the examined LVNC criteria at the post marathon visit. Captur criteria in this diagram is defined as a mean fractal dimension of >1.26.

Multiple validated methods of LV trabeculation measurement were assessed at baseline and after marathon training. Chin [9] and Jenni [10] criteria were assessed on echocardiography and Petersen [11], Jacquier [12] and Captur [13] criteria were assessed on CMR (Fig. 1). Stollberger criteria [14] were not satisfied at any time by any subject and therefore could not be used for quantitative comparison of LV trabeculation. No subject possessed > 3 trabeculations (long axis views) with color Doppler visualized perfusion of intertrabecular spaces. There was poor concordance between indices of trabeculation (Fig. 2). Jenni, Jacquier and Captur measurements did not change between baseline evaluation and after marathon training: Jenni ratio (0.89 vs. 0.89; *P* = 0.77), Jacquier trabeculated mass percentage (15.8 vs. 15.9; *P* = 0.76), Captur global mean LV fractal dimension (FD) (1.20 vs. 1.19; *P* = 0.37) and Captur maximum apical LV FD (1.36 vs. 1.36; *P* = 0.74). The Chin X/Y ratio demonstrated a small increase in trabeculation (0.56 vs. 0.52; *P* = 0.02), where 38% of the population were identified as excessively trabeculated at baseline, predominantly from apical measurements. The Petersen NC/C ratio showed a small reduction in trabeculation (1.21 vs. 0.92; *P* < 0.01), where excessive trabeculation (NC/C > 2.3) was seen in 3% at baseline and was unchanged after training. Seventy-three percent satisfied a Captur maximum apical FD > 1.3 at baseline, which indicated more apical trabecular complexity than expected but with no change in FD observed after marathon training.

Inter-observer agreement for Chin, Jenni, Petersen, Jacquier and Captur criteria was ICC = 0.63 (good), 0.22 (poor), 0.89 (excellent), 0.49 (fair) and 0.76 (excellent), respectively.

## 4 Discussion

This study found that LV trabeculation in novice marathon runners did not change after training for and running a first marathon. Despite little within-subject variability in LV trabeculation over the study period, methods of quantification for determining the prevalence of excessive LV trabeculation were highly discordant.

It has previously been shown that younger individuals possess greater amounts of apical trabeculation [15,16] but age-specific normative or cut-off values for pathological LV trabeculation do not currently exist. In this sample of healthy subjects, excessive trabeculation was found predominantly at the LV apex, which is recognized as the most commonly non-compacted segment [11] and was detected with greater sensitivity by Chin and Captur apical FD criteria. A higher prevalence of positive Chin compared to Jenni criteria has previously been reported in Olympic athletes with prominent trabeculation [2]. Exclusion of the LV apex is a requirement of some LVNC imaging criteria to improve test specificity [11,13], which may explain the low prevalence of positive Petersen criteria. The method and extent of apical exclusion inevitably introduces variation, in addition to measurement in different phases of the cardiac cycle and different imaging planes, accounting for poor concordance between LV trabeculation assessments described in this study and many others [14]. Based on these findings it may be argued that Stollberger criteria hold greater specificity for pathology but as we did not include subjects with suspected cardiomyopathy and it is currently unknown whether LVNC is indeed a distinct cardiomyopathy, this is speculative.

This work supports the findings of a large cross sectional study by Woodbridge et al. where they were unable to demonstrate a relationship between LV trabeculation and physical activity [17]. In their study, subjects with a mean age of 61 years had a median self-reported physical activity level equivalent to 4.4 h of running per week (2115 MET-min), which is less than the exposure of typical competitive athletes.

## 5 Limitations

In our study, subjects were advised to gradually increase running training to reach a peak exercise dose of 4.8 h per week, following a beginner's training plan, which is a modest exercise dose in comparison to competitive athletes. In addition, the absence of an increase in peak oxygen consumption may indicate that unsupervised exercise is an insufficient stimulus to induce the degree of athletic cardiac remodeling relevant to our hypothesis of increased LV trabeculation. A cross sectional study by Gati et al. compared young, competitive athletes (mean age 21 years) of various disciplines but not marathon runners, with a sedentary control population and demonstrated a higher prevalence of increased LV trabeculation (18.3% vs 7.0%), where the athletes exercised for an average of 17.7 h per week [1]. A longitudinal study involving a high-dose exercise stimulus and long interval imaging follow up would be required to investigate whether LV trabeculation might be induced by competitive athlete doses of exercise in predisposed individuals.

## 6 Conclusion

At present it is not clear whether high-dose exercise can induce trabeculation or expose concealed trabeculation. Additional challenges remain as methods of trabeculation quantification can be hindered by poor reproducibility and potential over-diagnosis of LVNC in many populations, including athletes. Automated methods of quantifying trabeculae [18,19] appeal as they overcome issues with reproducibility, have the potential to define normal ranges in healthy individuals from large CMR biobanks and reduce analysis time, which could facilitate better anatomical phenotyping. If currently applied thresholds fail to reliably differentiate healthy individuals from those with disease and do not provide information relating to prognosis [4], their value in current clinical practice is questionable.

## Ethics approval and consent to participate

Written consent was obtained from all participants and the National Research Ethics Service; Queen Square, London committee granted ethical approval (15/LO/086). The trial is registered on ClinicalTrials.gov, number NCT02568072.

## Funding

This work was jointly supported and funded by the 10.13039/501100000274British Heart Foundation with a clinical research training fellowship grant [FS/15/27/31465 to A.D.] and Cardiac Risk in the Young. This work was also supported by COSMED (Rome, Italy) through the provision of cardiopulmonary exercise testing equipment and technical support.

## CRediT authorship contribution statement

**Andrew D'Silva:** Writing - original draft, Conceptualization, Methodology, Formal analysis, Investigation, Resources, Data curation, Visualization, Project administration, Funding acquisition. **Gabriella Captur:** Conceptualization, Software, Validation, Formal analysis, Resources, Data curation, Writing - review & editing. **Anish N. Bhuva:** Validation, Writing - review & editing, Investigation. **Siana Jones:** Validation, Writing - review & editing, Investigation. **Rachel Bastiaenen:** Investigation, Writing - review & editing. **Amna Abdel-Gadir:** Investigation, Writing - review & editing. **Sabiha Gati:** Conceptualization, Methodology, Writing - review & editing. **Jet van Zalen:** Investigation, Writing - review & editing. **James Willis:** Investigation, Writing - review & editing. **Aneil Malhotra:** Writing - review & editing. **Irina Chis Ster:** Formal analysis, Supervision, Writing - review & editing. **Charlotte Manisty:** Supervision, Writing - review & editing. **Alun D. Hughes:** Supervision, Writing - review & editing. **Guy Lloyd:** Supervision, Writing - review & editing. **Rajan Sharma:** Supervision, Writing - review & editing. **James C. Moon:** Conceptualization, Methodology, Resources, Writing - review & editing, Supervision, Funding acquisition. **Sanjay Sharma:** Conceptualization, Methodology, Resources, Writing - review & editing, Supervision, Funding acquisition.

## Declaration of competing interest

The authors declare that the research was conducted in the absence of any commercial or financial relationships that could be construed as a potential conflict of interest. The study funders and supporters had no role in study design, data collection and analysis, decision to publish, or preparation of the manuscript.
